# Changes in hypothermal stress-induced hepatic mitochondrial metabolic patterns between fresh water- and seawater-acclimated milkfish, *Chanos chanos*

**DOI:** 10.1038/s41598-019-55055-4

**Published:** 2019-12-06

**Authors:** Chia-Hao Chang, Zong-Zheng Liu, Tsung-Han Lee

**Affiliations:** 1 Department of Life Sciences, National Chung Hsing University, Taichung, 402 Taiwan; 2 iEGG and Animal Biotechnology Center, National Chung Hsing University, Taichung, 402 Taiwan

**Keywords:** Energy metabolism, Animal physiology

## Abstract

Milkfish (*Chanos chanos*) is a tropical euryhaline species. It can acclimate to fresh water (FW) or seawater (SW) and be cultured in both. In winter, cold snaps cause huge losses in milkfish revenue. Compared to FW-acclimated individuals, SW-acclimated milkfish have better low-temperature tolerance. Under hypothermal stress, a stable energy supply is critical to maintain normal liver function. In this study, the levels of key mitochondrial enzymes (citrate synthase (CS) and cytochrome c oxidase (COX)) in milkfish livers were examined. The CS:COX activity ratio in FW milkfish significantly increased under hypothermal stress (18 °C) whereas ATP (the end product of aerobic metabolism) was downregulated. Therefore, the activities of the enzymes involved in mitochondrial amino acid biosynthesis (aspartate aminotransferase (AST) and glutamate dehydrogenase (GDH)) were evaluated to elucidate energy flow in milkfish livers under hypothermal stress. In FW milkfish, GDH activity was upregulated whereas AST activity was downregulated. Nevertheless, the levels of all the aforementioned enzymes did not significantly change in SW milkfish under hypothermal stress. In summary, we clarified the mechanism accounting for the fact that SW milkfish have superior low-temperature tolerance to FW milkfish and demonstrated that SW and FW milkfish have different and unique strategies for regulating energy flow.

## Introduction

Fish are ectotherms. Cold stress causes a rapid reduction in their body temperature and affects their physiological and behavioural responses. Environmental temperature significantly influences biochemical activity in ectotherms. The ambient temperature affects the rates of enzymatic reactions and substrate diffusion. It can also alter membrane fluidity^1,2^. Recent studies on teleosts identified both their upper and lower thermal tolerance range limits^3,4^. Internal hypoxia and metabolic depression were observed in fish outside their optimal temperature ranges^2,5^. These responses occurred because the cold water reduced the capacities of oxygen uptake and delivery, although dissolved oxygen was increased in cooler water. Oxygen demands of the fish were not met because the mechanisms regulating O_2_ absorption were inhibited^6,7^.

Aerobic energy production is correlated with mitochondrial metabolism. This process consists of the citric acid (TCA; Krebs) cycle and the electron transport chain (ETC), and it synthesises most of the adenosine triphosphate (ATP) in an organism. Aerobic metabolism activity can be estimated from oxidative enzymes including citrate synthase (CS) and cytochrome c oxidase (COX), the crucial indicator enzymes of the TCA cycle and the ETC, respectively^8,9^. The TCA cycle generates NADH which is used for ATP production process in the ETC. The TCA cycle also produces intermediates which are precursors of non-essential amino acids^10^. Hypothermal stress triggers compensatory physiological responses such as mitochondrial proliferation and aerobic enzyme upregulation. These effects have been extensively studied in teleosts^11–16^. Mitochondrial density and hepatocyte volume were increased in the livers of the blenny (*Blennius pholis*) and the threespine stickleback (*Gasterosteus aculeatus*)^11,12^. In the livers of temperate-zone cod (*Gadus morhua*), eelpout (*Zoarces viviparous*), and threespine stickleback, CS activities in livers were upregulated in cold environments whereas COX activity did not change^13–15^. Similar enzyme function patterns were observed in the subtropical species gilthead sea bream after cold acclimation^16^. In all of these teleosts, the CS:COX activity ratios were elevated under cold stress^13–16^. However, the metabolic patterns in mitochondria of fish livers under hypothermal stress have never been elucidated.

TCA cycle also plays the role of amino acid biosynthesis. The oxaloacetate and α-ketoglutarate are crucial amino acid biosynthesis intermediates. Glutamate dehydrogenase (GDH) and aspartate aminotransferase (AST) regulate amino acid biosynthesis and glutamate deamination, respectively, and are indicators of hepatic damage^17,18^. AST activity has been widely used as a plasmatic indicator of liver injury. Nevertheless, there are comparatively few studies on amino acid biosynthesis in the same context. High-protein soybean meal downregulated AST in the livers of juvenile Japanese seabass (*Lateolabrax japonicus*) because it exogenously supplied them with abundant quantities of all essential amino acids^19^. On the other hand, the direction of the GDH reaction varies with each specific tissue/organ. In the liver, the GDH reaction is driven by a bidirectional equilibrium. The direction of the reaction is determined by relative substrate concentrations and coenzyme ratios^18,20^.

Milkfish (*Chanos chanos*) are euryhaline, stenothermic teleosts. They are distributed in the tropical and subtropical waters of the Indo-Pacific Ocean. They can be cultured in environments ranging from fresh water (FW) to seawater (SW)^21^. This species is important for aquaculture in Southeast Asian countries. In Taiwan, the average temperature in summer and winter is 28 °C and 18 °C, respectively. Being a tropical species, sometimes the cold snaps (below 10 °C) in the wintertime caused high mortality rate of milkfish and resulted in huge yield and economic losses in Taiwan^22^. Previous studies have shown that compared to FW-acclimated ones, SW-acclimated milkfish had better low-temperature tolerance^23^. Under hypothermal stress (18 °C), glycogen breakdown increased in FW milkfish livers to maintain blood glucose levels. In contrast, this response was not observed in SW milkfish^24^. Lactate dehydrogenase (LDH) is an indicator of hepatic gluconeogenesis identified by the two-dimensional gel electrophoresis of SW milkfish livers^25^. Transcriptomic analyses of the metabolic pathways in the brains, gills, livers, and kidneys of hypothermal-stressed milkfish generated fragments per kilobase of transcript per million reads mapped (FPKM) profiles which revealed that oxidative phosphorylation (relative genes of Complex I-V complex) and catabolism genes (fumarylacetoacetase, glutamate dehydrogenase, 3-methlcrotonyl-CoA carboxylase, phenylalanine hydroxylase, and pyruvate carboxylase) were upregulated to increase energy production in SW milkfish but not in FW milkfish^26^. Therefore, transcriptomic and proteomic analyses provided new insights into the energy and mitochondrial metabolism of milkfish under low-temperature stress. In addition, different cold tolerance ability between FW and SW milkfish^23^ suggested that the roles of their livers, the energy supply and de-toxic organ, might be salinity-dependent under hypothermal stress. Glycogen breakdown differed between SW- and FW-acclimated milkfish under hypothermal stress^24^ also implied that the metabolic patterns in their hepatic mitochondria at low temperatures varied with environmental salinities. To elucidate the roles of liver mitochondria in maintaining energy supply in response to low-temperature stress, oxygen consumption was measured in SW- and FW-acclimated milkfish subjected to 18 °C. Indicators of mitochondrial aerobic metabolism and amino acid biosynthesis were also compared between SW and FW milkfish under hypothermal stress.

## Materials and Methods

### Fish and experimental conditions

Juvenile milkfish were purchased from a local fish farm in Changhua, Taiwan. The environmental salinity was ~3‰. The fish were maintained for ≥1 month in 400-L tanks containing FW and SW (35‰) at 28.0 ± 0.5 °C, respectively. The photoperiod was 12/12 h light/dark. SW was prepared from local tap water by adding appropriate amounts of reef sea salt (Qingdao Sea-Salt Aquarium Technology, Qingdao, China). Fresh water and artificial seawater were continuously recirculated through fabric-floss filters and partially replaced every month. The fish were fed daily with commercial pellets. For hypothermal treatments, six FW-acclimated and six SW-acclimated milkfish were transferred to the cooling tank with FW and SW, respectively. The recirculated FW or SW in the cooling tank was cooled at a constant rate (2 °C h^−1^) from 28 °C to 18 °C using a coolant system (PF-225M, PRINCE, Tainan, Taiwan). Then the FW and SW milkfish were kept at 18 °C for one week as the hypothermal groups. Meanwhile, for the control group, another six FW and six SW milkfish were transferred to the other tanks with recirculated FW and SW at 28 °C. Subsequently, the fish (average total length: 10.2 ± 0.2 cm; average body weight: 13.7 ± 1.2 g) were anesthetised with 0.5% 2-phenoxyethanol then sacrificed by cutting their spinal cords. The fish experimentation protocol was reviewed and approved by the Institutional Animal Care and Use Committee (IACUC) of the National Chung Hsing University, Taichung, Taiwan (IACUC Approval No. 105-024 to THL). All experiments were carried out in accordance with the approved guidelines.

### Respiratory frequency (*f*_R_)

Experiments were performed according to the method of Hardewig *et al*.^27^ with certain modifications. The fish were incubated in 100 L FW and SW tanks fitted with the coolant system, respectively. Water temperatures were maintained either at 28 °C (control group) or 18 °C (study group). Respiratory frequency was determined after 1-week acclimation by counting the opercular movements of each individual in 5 min (three replicates, n = 8).

### Oxygen consumption rate (OCR)

The oxygen consumption rate (OCR) was analysed according to the method of Del Toro-Silva *et al*.^28^ with some modifications. The circular sealed plastic chamber (diameter, 25 cm; height, 15 cm; volume, 5.0 L), constructed by Dr. Cheng-Hao Tang, was consisted of a removable plastic lid including a hole with airtight rubber that can set the dissolved oxygen (DO) meter (YSI 52, Yellow Springs Instruments, Yellow Springs, OH, USA) to detect DO in water. The chamber was placed in the water bath (B601D, Firstek, New Taipei City, Taiwan) either at normal (28.0 ± 0.5 °C) or hypothermal (18.0 ± 0.5 °C) water temperature. Then one milkfish was transferred to the chamber and kept for 30 min to be stable in behavior, but not in continuous swimming, before analysis. Six milkfish (average total length: 10.5 ± 0.7 cm; average body weight: 13.0 ± 1.8 g) per treatment were separately stored in a sealed plastic chamber. The water in chamber was renewed per experiment to reduce the effects of metabolites^29,30^. The oxygen consumption rate per individual was measured every 10 min for 1 h. The formula for OCR (O_2_ kg^−1^ h^−1^) is: [O_2·T1_ (mg) − O_2·T2_ (mg)/10 (min) × 60 (h)/fish body weight (kg)].

### Quantitative Real-Time (qRT) PCR

The qRT-PCR was performed according to Chang *et al*.^31^. The RNAspin Mini RNA Isolation Kit (GE Health Care, Piscataway, NJ, USA) was used to extract sample RNA and prevent it from being contaminated by genomic DNA. The quality of the extracted RNA was determined by the A260/A280 ratio (2.0–2.2) using NanoDrop 2000 (Thermo Fisher Scientific, Wilmington, CA, USA) and by RNA gel electrophoresis. First-strand cDNA was synthesised from 1 μg of total RNA using the iScript Reverse Transcription Supermix (Bio-Rad Laboratories, Hercules, CA, USA). Partial sequences of the milkfish transcriptomic database were used to design the primer sequence (Table 1) for qRT-PCR with Primer3Plus (http://primer3plus.com/cgi-bin/dev/primer3plus.cgi). They were based on highly conserved regions compared with those of other teleosts. Primer amplification efficiencies were predicted to be 90–105%. The qRT-PCR mixture contained 8 μL cDNA (100 × dilution), 2 μL of 1 μM primers, and 10 μL of 2 × KAPA SYBR FAST qPCR Kit Master Mix (Kapa Biosystems, Boston, MA, USA). The relative gene expression was analysed by the comparative Ct method using the formula 2^−[(Cttarget gene, n − Ctef1a, n) − (Cttarget gene, control − Ctef1a, control)]^ where Ct is the threshold cycle number^32^. All liver cDNA sample mixtures were used as running controls. For each cDNA sample, independent assays were performed in triplicate.

**Table 1:** Primers used for qRT-PCR.

Primer	Sequence (5′-3′)
*Cccox4*qF158	GAGAGCTTTGCAGAGATGAACC
*Cccox4*qR242	GCCCAGTGAAGCCAATGAAG
*Cccs*qF1038	GCAGGGCAAGGCCAAGAACC
*Cccs*qR1153	GCGGGACAACACAAACAGCAC
*Ccef1α*qF	CCATTGTTCAGATGATTCCCG
*Ccef1α*qR	CTTCTTGATGACACCACCAGC

### Immunoblotting

Immunoblotting was performed according to the method of Chang *et al*.^33^. Aliquots containing 40 μg of hepatic homogenates were heated at 95 °C for 5 min with 6× sample buffer. They were then fractionated by electrophoresis on sodium dodecyl sulphate (SDS) containing 14% polyacrylamide gel. Pre-stained protein molecular weight marker (#26616, Thermo Fisher Scientific, Waltham, MA, USA) was applied in the electrophoresis. The fractionated proteins were transferred to 0.22-μm polyvinylidene difluoride (PVDF) blotting membranes (Millipore, Bedford, MA, USA). Membranes were pre-incubated for 1 h in phosphate-buffered saline with Tween-20 (PBST) with 5% (w/v) nonfat dried milk to minimise nonspecific binding. Blots were incubated overnight at 4 °C with primary antibodies including COX subunit 4 monoclonal antibody (1:3,000 dilution; #4850; Cell Signaling Technology, Beverly, MA, USA), citrate synthase monoclonal antibody (1:3,000 dilution; sc-390693; Santa Cruz Biotechnology Inc., Dallas, TX, USA), and β-actin polyclonal antibody (1:5,000 dilution; GTX109639; GeneTex, Irvine, CA, USA). They were then incubated at 28 °C for 1 h with HRP-conjugated secondary antibodies including goat anti-rabbit IgG (GTX23110-01; GeneTex) for the primary antibodies of COX subunit 4 and citrate synthase and rabbit anti-mouse IgG (GTX213112-01; Genetex) for the primary antibody of β-actin. The western chemiluminescent HRP substrate (EMD Millipore, Billerica, MA, USA) was used to develop the immunoblots. Images were photographed with a cool-charge-coupled device (CCD) camera (ChemiDoc XRS^+^; Bio-Rad) and analysed with Image Lab v. 3.0 (Bio-Rad) to compare the numerical values with the relative immunoreactive band intensities.

### Enzyme activity

The experimental fish were anesthetized with 0.5% 2-phenoxethanol before sampling. Milkfish livers were dissected quickly, immersed in liquid nitrogen, and stored at −80 °C for less than one week before analyses of following enzyme activities.

#### Citrate Synthase (CS) Activity (EC 2.3.3.1)

Frozen fish liver was homogenised in ice-cold SEI buffer (150 mM sucrose, 10 mM EDTA, and 50 mM imidazole; pH 7.5) and proteinase inhibitor (v/v: 25/1; Roche Diagnostics, Risch-Rotkreuz, Switzerland) in a Polytron PT1200E (Kinematica, Bohemia, NY, USA) for 10 sec at maximum speed. The homogenates were centrifuged at 1,500 × *g* and 4 °C for 10 min. The supernatants were centrifuged at 13,000 × *g* and 4 °C for 10 min. The pellets were resuspended in SEI buffer containing proteinase inhibitor. Mitochondrial fraction samples were diluted to 50 μL with CS assay buffer following the manufacturer’s instructions (#K318-100, Biovision, Milpitas, CA, USA). Glutathione was quantified with a standard curve. A 96-well microplate was analysed in kinetic mode at 28 °C for 20 min using a VERSAmax microplate reader (Molecular Devices LLC, San Jose, CA, USA) at 412 nm. The protein concentrations of the supernatants were determined with the reagents in a protein assay kit (Bio-Rad). Bovine serum albumin (BSA; Sigma-Aldrich Corp., St. Louis, MO, USA) was used as a standard. CS activity = B/(ΔT·V)·D mg protein, where B is the number of nanomoles of S-H group from the standard curve, ΔT is the reaction time (min), V is the sample volume added to the reaction well, and D is the dilution factor.

#### Cyclooxygenase (COX) activity (EC 1.9.3.1)

The decrease in OD_550_ (optical density at 550 nm) based on cytochrome c oxidation was used to calculate COX activity. The mitochondrial fractions were processed as described above. A 10-μL sample of mitochondrial fraction alone was used as a positive control, and the negative control did not contain mitochondrial fraction. All subsequent steps were performed according to the manufacturer’s instructions (#K287-100, Biovision). A 96-well microplate was analysed in kinetic mode at 28 °C for 5 min in a VERSAmax microplate reader (Molecular Devices LLC) at 550 nm. The protein concentrations of the supernatants were determined with reagents from a protein assay kit (Bio-Rad). Bovine serum albumin (BSA; Sigma-Aldrich Corp.) was used as a standard. COX activity (U mg^−1^) = ΔOD_550_/Time (Δt)/(7.04 × mg protein), where ΔOD_550_ is the difference in OD between time (t1) and time (t2). Δt is the difference between time t1-t2 (min).

#### Glutamate dehydrogenase (GDH) activity (EC 1.4.1.2)

GDH activity was evaluated from the decrease in OD_450_ as a result of NADH oxidation. One hundred milligrams of liver tissue homogenate was placed in 500 μL GDH assay buffer and all subsequent steps were performed according to the manufacturer’s instructions (#K729−100, Biovision) with some modifications. The reaction mixture contained 1 M α-ketoglutarate, 7.5 mM NADH, and GDH Developer (#K729-100-3, Biovision). The 50 μL samples and reaction mixture were incubated at 28 °C and analysed in a VERSAmax microplate reader (Molecular Devices LLC) at 450 nm. GDH activity (mU mg^−1^) = B/(ΔT·V)/g wet wt, where B is amount of NADH in nmol calculated from the standard curve, ΔT is the reaction time (in min), and V is sample volume in mL added to the reaction well.

#### Aspartate aminotransferase (AST) activity (EC 2.6.1.1)

AST activity was used to calculate glutamate deamination at OD_450_. One hundred milligrams of liver tissue homogenate was placed in 500 μL AST assay buffer and all subsequent steps were performed according to the manufacturer’s instructions (K753-100, Biovision). Serial glutamate dilutions (0 nmol, 2 nmol, 4 nmol, 6 nmol, 8 nmol, and 10 nmol in 50 μL assay buffer) were used to plot the standard curve. The 50 μL samples were incubated at 28 °C and analysed in a VERSAmax microplate reader (Molecular Devices LLC) at 450 nm. AST activity (mU mg^−1^) = B/((T_2_ − T_1_)·V)/g wet wt, where B is the amount of glutamate in nmol calculated from the standard curve, T_1_ is the time of the first reading (in min), and T_2_ is the time of the second reading (in min).

### ATP content

The frozen liver tissues were weighed and homogenised in ice-cold SEI buffer (150 mM sucrose, 10 mM EDTA, and 50 mM imidazole, pH 7.5) with a Polytron PT1200E (Kinematica) for 10 sec at maximum speed. Since the tissue samples contained enzymes which could rapidly consume ATP, perchloric acid (PCA) was added to denature most of protein present. The homogenates were centrifuged at 5,000 × *g* and 4 °C for 5 min. Then 500 μL supernatants were mixed with 100 μL ice-cold 4 M PCA for deproteinisation, incubated at 4 °C for 5 min, and centrifuged at 13,000 × *g* and 4 °C for 2 min. After deproteinisation, the supernatants were neutralised with 20 μL ice-cold 2 M KOH at 4 °C for 5 min. All subsequent steps were done according to the manufacturer’s instructions (#K354-100, Biovision). Serial ATP dilutions (0 nmol, 2 nmol, 4 nmol, 6 nmol, 8 nmol, and 10 nmol/well) were used to plot the standard curve. Absorbances were measured in a VERSAmax microplate reader (Molecular Devices LLC) at 570 nm. Sample ATP contents were determined from the standard curve.

### Statistical analysis

Values were expressed as means ± SEM (standard error of the mean) and compared by two-way ANOVA with Tukey’s HSD post-hoc method in R v. 3.4.2 (R Foundation, Vienna, Austria). *P* < 0.05 was set as the significance level.

## Results

### Respiratory frequency (*f*_R_) and oxygen consumption rate (OCR)

**Figure 1:**
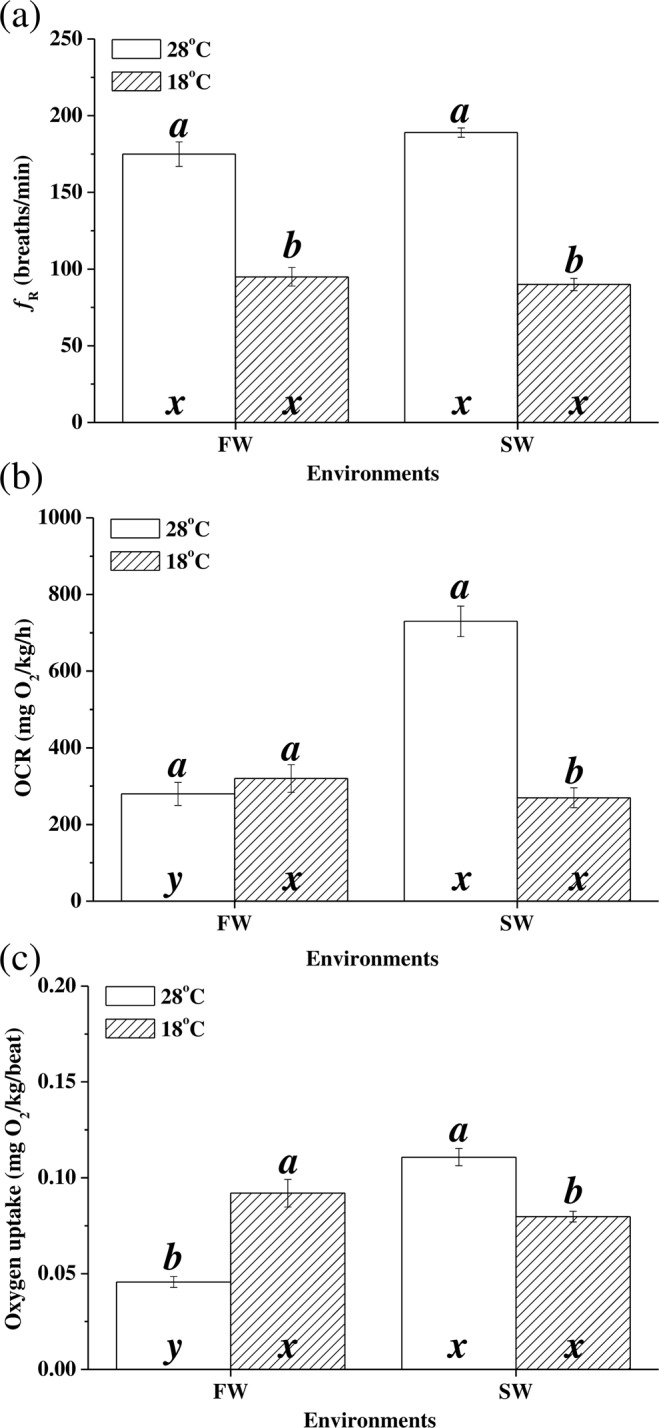
Respiratory frequency (**a**), oxygen consumption rate (**b**), and oxygen uptake rate (**c**) in milkfish acclimated to 28 °C (white bar) and 18 °C (striped bar), respectively. Different letters (**a,b**) indicate significant differences between the 28 °C and 18 °C groups. The x and y indicate significant differences between the FW and SW groups according to Tukey’s HSD pairwise comparison followed by two-way ANOVA (Table 2). Values are means ± SEM, n = 8. *f*_R_: respiratory frequency; OCR: oxygen consumption rate; FW, fresh water; SW, seawater.

Temperatures changes significantly affected *f*_R_ according to two-way ANOVA (Table 2). The average *f*_R_ at 28 °C were 173 ± 7 and 187 ± 4 breaths min^−1^ for the FW and SW milkfish, respectively. At 18 °C, the average *f*_R_ significantly decreased by almost half for both FW (92 ± 7 breaths min^−1^, p < 0.001) and SW milkfish (93 ± 6 breaths min^−1^, p < 0.001) (Fig. 1a).

**Table 2:** Two-way ANOVA of the effects of temperature and salinity on the livers of milkfish.

	Temperature			Salinity			Temperature x Salinity		
	F	df	P	F	df	P	F	df	P
*f*_R_	86.34	1, 28	<0.01^**^	0.001	1, 28	0.99	1.37	1, 28	0.25
OCR	0.23	1, 28	0.64	0.006	1, 28	0.94	20.29	1, 28	<0.01^**^
Oxygen uptake rate	48.75	1, 28	<0.01^**^	3.3679	1, 28	0.08	67.93	1, 28	<0.01^**^
*cs* mRNA	0.19	1, 20	0.66	2.30	1, 20	0.14	2.10	1, 20	0.16
*cox4* mRNA	0.103	1, 20	0.10	1.33	1, 20	0.26	2.13	1, 20	0.16
CS protein	0.31	1, 20	0.59	23.26	1, 20	<0.01^**^	2.13	1, 20	0.16
COX4 protein	18.45	1, 20	<0.01^**^	25.85	1, 20	<0.01^**^	4.26	1, 20	0.05^*^
CS activity	13.49	2, 30	<0.01^**^	39.70	1, 30	<0.01^**^	3.73	2, 30	0.04^*^
COX activity	2.34	2, 30	<0.01^**^	3.12	1, 30	0.08	2.79	2, 30	0.07
CS/COX	7.93	2, 30	<0.01^**^	2.72	2, 30	0.11	11.10	2, 30	0.02^*^
ATP content	26.71	1, 28	<0.01^**^	25.19	1, 28	<0.01^**^	11.08	1, 28	<0.01^**^
GDH activity	20,80	2, 30	0.01^**^	1.05	1, 30	0.31	4.15	2, 30	0.02^*^
AST activity	25.53	2, 36	<0.01^**^	1.69	1, 36	0.20	1.41	2, 36	0.26

Abbreviations: *f*_R_: respiratory frequency; OCR: oxygen consumption rate; CS, citrate synthase; COX, cytochrome c oxidase; *gdh*: glutamate dehydrogenase; *aatm*: aspartate aminotransferase, mitochondrial form; AST: aspartate aminotransferase; df = degree of freedom, F = F statistic, ^*^P ≤ 0.05; ^**^P ≤ 0.01. Values in bold indicate significant differences.

The OCR of SW milkfish (721 ± 51 mg O_2_ kg^−1^ h^−1^) at 28 °C was significantly higher than that of FW milkfish (291 ± 28 mg O_2_ kg^−1^ h^−1^; p < 0.001). Under hypothermal stress (18 °C), the OCR of the FW group did not significantly change (300 ± 44 mg O_2_ kg^−1^ h^−1^; p = 0.919). For the SW milkfish, however, OCR significantly decreased from 721 ± 51 mg O_2_ kg^−1^ h^−1^ to 282 ± 19 O_2_ kg^−1^ h^−1^ (p < 0.001; Fig. 1b). According to the two-way ANOVA, OCR was affected by the synergistic interaction between salinity and temperature (Table 2).

The oxygen uptake rate is the ratio of oxygen consumption to gill ventilation frequency per breath. At 28 °C, the oxygen uptake rate in SW milkfish (6.65 ± 0.27 mg O_2_ kg^−1^ breath^−1^) was significantly higher than that in FW milkfish (2.74 ± 0.17 O_2_ kg^−1^ breath^−1^; p < 0.001). At 18 °C, the oxygen uptake in FW milkfish increased to 5.52 ± 0.43 mg O_2_ kg^−1^ breath^−1^ (p < 0.001) whereas in SW milkfish it had decreased to 4.79 ± 0.17 mg O_2_ kg^−1^ breath^−1^ (p < 0.001; Fig. 1c). Two-way ANOVA showed that the oxygen uptake rate was also affected by synergistic interaction between temperature and salinity (Table 2).

### Low-temperature effects on liver *Cccs* and *Cccox4* expression

**Figure 2:**
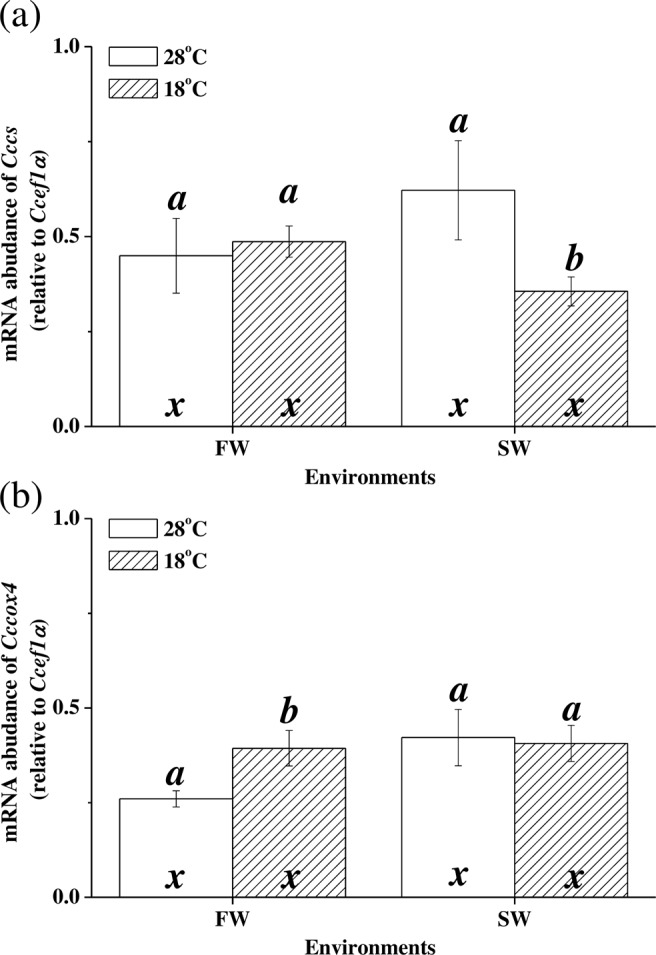
Expression of hepatic *Cccs* (**a**) and *Cccox4* (**b**) in milkfish acclimated to 28 °C (white bar) and 18 °C (striped bar). Different letters (**a**,**b**) indicate significant differences between the 28 °C and 18 °C groups. The x indicates no significant difference between the FW and SW groups according to Tukey’s HSD pairwise comparison followed by two-way ANOVA (Table 2). Values are means ± SEM, n = 6. FW, fresh water; SW, seawater.

At 18 °C, the mRNA expression of *Cccs* was downregulated in SW milkfish whereas it did not significantly change for FW milkfish (p = 0.740; Fig. 2a). At 18 °C, the expression of *Cccs* was significantly lower in the SW group than in the FW group (p = 0.029). On the other hand, hypothermal stress upregulated mRNA expression of *Cccox4* in FW milkfish (p = 0.036) but had no significant effect on that for the SW milkfish (p = 0.863; Fig. 2b). Two-way ANOVA demonstrated that the mRNA expression levels of *Cccs* and *Cccox4* were not significantly influenced by temperature or salinity (Table 2).

### Protein expression levels of Cccs and Cccox4 under hypothermal stress

**Figure 3:**
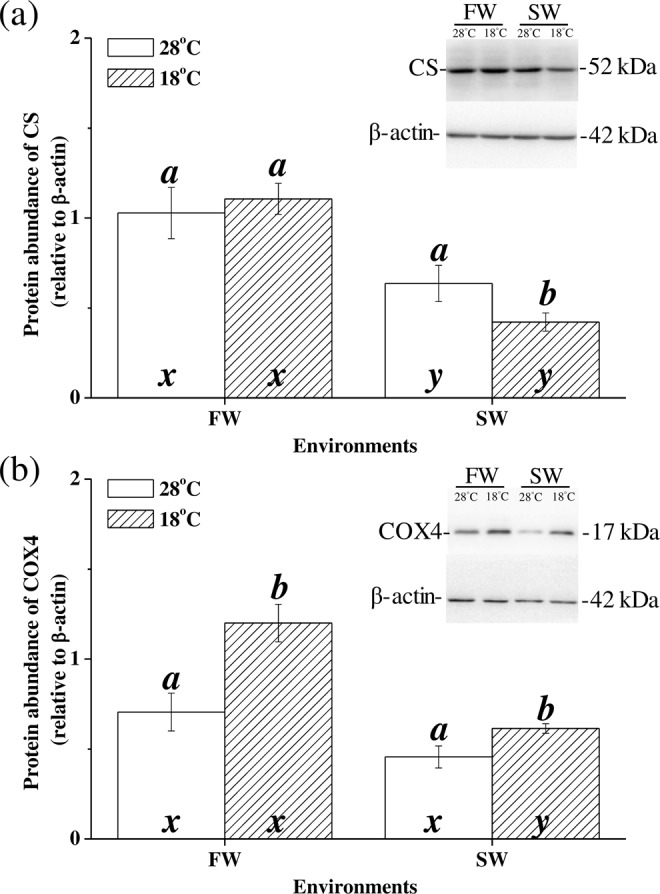
Relative protein abundance of hepatic citrate synthase (CS) and cytochrome c oxidase subunit 4 (COX4) in milkfish acclimated to 28 °C (white bar) and 18 °C (striped bar). Representative immunoblots of CS (**a**) and COX4 (**b**) show a single immunoreactive band. Different letters (**a,b**) indicate significant differences between the 28 °C and 18 °C groups. The x and y indicate significant differences between the FW and SW groups according to Tukey’s HSD pairwise comparison followed by two-way ANOVA (Table 2). Values are means ± SEM, n = 6. FW, fresh water; SW, seawater.

The immunoblots disclosed the CcCS and CcCOX4 proteins as single immunoreactive bands at 52 kDa and 17 kDa, respectively. The relative CcCS protein expression in SW milkfish was downregulated at 18 °C (p = 0.046; Fig. 3a). According to two-way ANOVA, salinity significantly influenced CcCS protein expression (Table 2). The relative CcCOX4 protein expressions in both SW and FW milkfish were upregulated under hypothermal stress (p = 0.050 and p = 0.076, respectively; Fig. 3b). Two-way ANOVA indicated that CcCOX4 protein expression was influenced either by low temperature or by salinity stress (Table 2).

### Changes in CS and COX Activity under hypothermal stress

**Figure 4:**
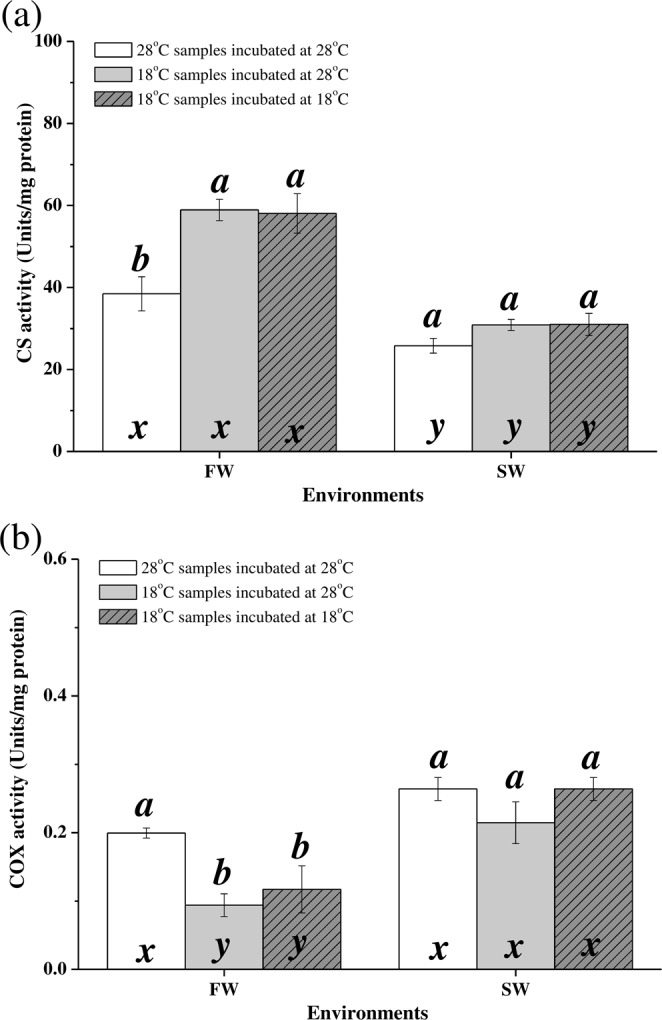
Activity levels of CS (**a**) and COX (**b**) in the livers of milkfish acclimated to 28 °C and 18 °C. Different letters (**a,b**) indicate significant differences between the 28 °C and 18 °C groups. The x and y indicate significant differences between the FW and SW groups according to Tukey’s HSD pairwise comparison followed by two-way ANOVA (Table 2). Values are means ± SEM, n = 6. FW, fresh water; SW, seawater.

Under hypothermal stress, CS activity was upregulated in FW milkfish (p = 0.003) but did not significantly change in SW milkfish (p = 0.068; Fig. 4a). The CS activities of low-temperature samples assayed at 28 °C or 18 °C in FW and SW individuals were not significantly different. Two-way ANOVA showed that both the synergistic interaction between temperature and salinity and each factor alone significantly affected CS activity (Table 2). The COX activity in FW milkfish was downregulated by hypothermal stress (p < 0.001) whereas it had no significant effect on COX activity in SW milkfish (p = 0.057). The COX activities of low-temperature samples assayed at 28 °C or 18 °C in FW and SW individuals were not significantly different. The COX activity of SW milkfish was higher than that of FW milkfish at 18 °C (p = 0.032; Fig. 4b). Two-way ANOVA demonstrated that either temperature or salinity factor significantly affects COX activity (Table 2).

**Figure 5:**
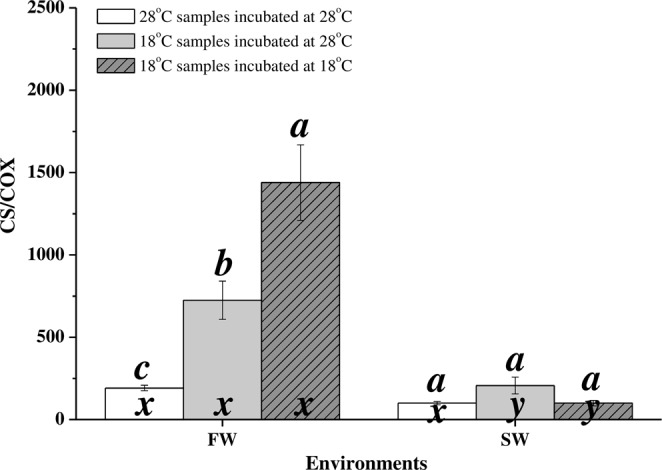
CS:COX activity ratios in milkfish livers. Different letters (**a–c**) indicate significant differences between the 28 °C and 18 °C groups. The x and y indicate significant differences between the FW and SW groups HSD pairwise comparison followed by two-way ANOVA (Table 2). Values are means ± SEM, n = 6. FW, fresh water; SW, seawater.

At 18 °C, the CS:COX activity ratio was significantly higher in FW milkfish than in SW milkfish (p = 0.005). The CS:COX activity ratio was upregulated in FW milkfish under hypothermal stress (p = 0.005) whereas it did not significantly change in SW milkfish (p = 0.093; Fig. 5). The CS:COX activity ratio of low-temperature samples assayed at 18 °C in FW individuals were enhanced more than those assayed at 28 °C. According to two-way ANOVA, the CS:COX activity ratio was significantly influenced by the synergistic interaction between salinity and temperature (Table 2).

### ATP content

**Figure 6:**
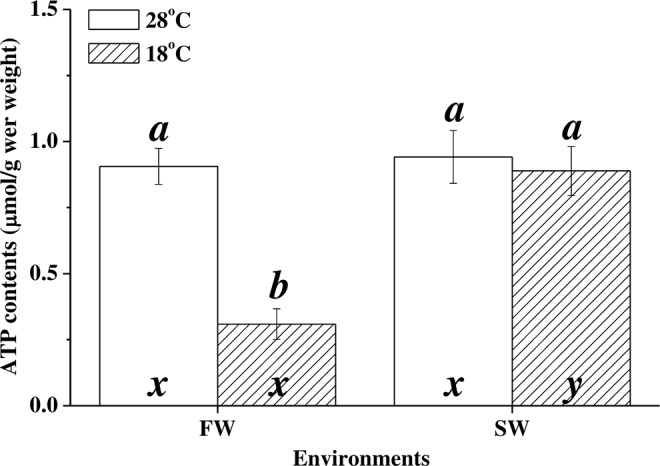
ATP levels in the livers of milkfish acclimated to 28 °C (white bar) and 18 °C (striped bar). Different letters (**a,b**) indicate significant differences between the 28 °C and 18 °C groups. The x and y indicate significant differences between the FW and SW groups according to Tukey’s HSD pairwise comparison followed by two-way ANOVA (Table 2). Values are means ± SEM, n = 8. FW, fresh water; SW, seawater.

The ATP level in the FW milkfish livers was 0.906 ± 0.068 μmol g^−1^ wet weight at 28 °C and significantly decreased to 0.309 ± 0.058 μmol g^−1^ wet weight at 18 °C (p < 0.001). However, the ATP content in the livers of the SW milkfish did not significantly differ with temperature (28 °C group vs. 18 °C group: 0.942 ± 0.100 μmol g^−1^ wet weight vs. 0.889 ± 0.093 μmol g^−1^ wet weight; p = 0.703) (Fig. 6). Two-way ANOVA showed that there was synergistic interaction between salinity and temperature and it significantly affected the ATP levels in milkfish livers (Table 2).

### Indicators of the amino acid biosynthesis pathway in the TCA cycle

**Figure 7:**
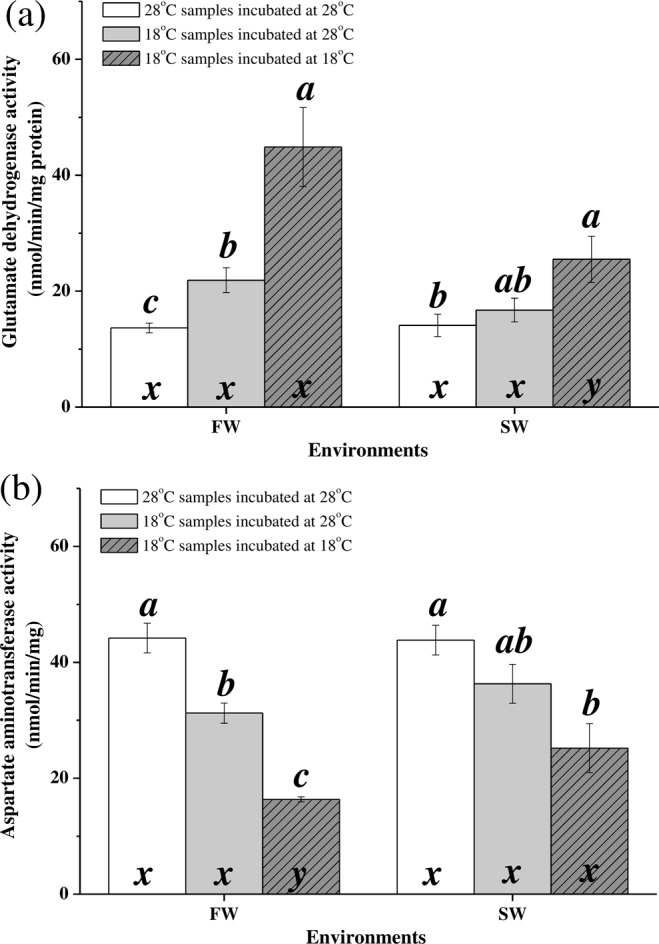
Activities of (**a**) glutamate dehydrogenase and (**b**) aspartate aminotransferase in the livers of milkfish acclimated to 28 °C and 18 °C. Different letters (**a–c**) indicate significant differences between the 28 °C and 18 °C groups. The x and y indicate significant differences between the FW and SW groups according to Tukey’s HSD pairwise comparison followed by two-way ANOVA (Table 2). Values are means ± SEM, n = 8. FW, fresh water; SW, seawater.

Under hypothermal stress, glutamate dehydrogenase (GDH) activity was upregulated in FW milkfish (28 °C group vs. 18 °C group assayed at 28 °C: 13.66 ± 0.84 nmol min^−1^ mg^−1^ vs. 21.89 ± 2.12 nmol min^−1^ mg^−1^, p = 0.009), and samples assayed at 18 °C (44.87 ± 6.80 nmol min^−1^, p = 0.001) were significantly increased than those assayed at 28 °C. In SW milkfish, however, GDH activity did not significantly change with temperature (28 °C group vs. 18 °C group: 14.12 ± 1.93 nmol min^−1^ mg^−1^ vs. 16.74 ± 2.07 nmol min^−1^ mg^−1^, p = 0.374). The activities of samples of SW18 °C group assayed at 18 °C (25.49 ± 3.99 nmol min^−1^, p = 0.001) was not significantly different compared to those assayed at 28 °C, but significantly increased than those of the SW28 °C group (p = 0.023) (Fig. 7a). GDH activity was significantly affected by temperature (Table 2). The aspartate aminotransferase (AST) activity was downregulated in FW milkfish (28 °C group vs. 18 °C group: 44.19 ± 2.56 nmol min^−1^ mg^−1^ vs. 31.24 ± 1.75 nmol min^−1^ mg^−1^; p = 0.002) whereas low-temperature samples assayed at 18 °C was reduced than the 28 °C samples (p = 0.001). Moreover, the AST activity did not significantly change with temperature in SW milkfish (28 °C group vs. 18 °C group: 43.84 ± 2.56 nmol min^−1^ mg^−1^ vs. 36.30 ± 3.37 nmol min^−1^ mg^−1^; p = 0.101), and low-temperature samples assayed at 18 °C (25.20 ± 4.21 nmol min^−1^ mg^−1^) was significantly down-regulated than the SW28 °C group (p = 0.003) (Fig. 7b). The AST activity was significantly affected only by temperature (Table 2).

## Discussion

### Oxygen absorption under hypothermal stress

In the present study, the respiratory frequency (*f*_R_) and oxygen consumption rate (OCR) under low-temperature stress indicated that milkfish are “oxygen regulators” like Amur sturgeon (*Acipenser schrenckii*)^34^, grass carp (*Ctenopharyngodon idella*)^35^, goldfish (*Carassius auratus*)^36^, and tilapia (*Oreochromis niloticus*)^37^. At 28 °C, OCR of SW milkfish were significantly higher than those of FW milkfish because SW milkfish have relatively higher swimming and routine activity levels^38^. The *f*_R_, OCR, and oxygen uptake rate per breath of SW milkfish were downregulated in response to hypothermal stress as they were for the other aforementioned species. On the other hand, although *f*_R_ was downregulated in FW milkfish under hypothermal stress, the OCR pattern was consistent. Therefore, the oxygen uptake capacity of FW milkfish gills could be regulated under low-temperature stress. Both environmental temperature and salinity affect dissolved oxygen levels. Low temperatures and hypoosmosis enhanced oxygen solubility^39^. Although DO is higher in low-temperature than in normal-temperature environments, the oxygen uptake rates (OCR/*f*_R_) of the FW/18 °C group suggest that these fish enhance oxygen absorption in their gills.

### Responses of aerobic metabolism to hypothermal stress

Several teleosts undergo compensatory changes in CS activity under hypothermal stress. Upregulation of CS activity and consistency in COX activity were found in the livers of cod (*Gadus morhua*)^14^, eelpout (*Zoarces viviparous*)^13,15^, gilthead sea bream (*Sparus aurata*)^16^, and threespine stickleback (*Gasterosteus aculeatus*)^12^ under hypothermal stress. In contrast, the COX activity levels in the livers of goldfish (*Carassius auratus*)^40^ were downregulated whereas the CS and COX activity levels remained constant in the livers of lake whitefish (*Coregonus clupeaformis*)^41^. The CS:COX activity ratio reflects anabolic demand in mitochondria of livers under hypothermal stress. TCA cycle intermediates are utilised to produce non-essential amino acids, fatty acids, and nucleotides. In the eelpout, the ratio of CS:COX activities were upregulated in response to cold adaptation in the Baltic- and North Seas^15^. The COX activity and CS activity were suggested to be related to changes in mitochondrial membrane structure and mitochondrial matrix volume^12,42^. However, the functions of ratio of CS:COX activities have not been clearly described in the other teleosts, and the anabolic indicators in the TCA cycle remain unclear. In the present study, the CS activities in FW-acclimated milkfish were upregulated but the COX activities were downregulated under hypothermal stress. The CS:COX ratio in FW-acclimated milkfish significantly increased under hypothermal stress. Therefore, the anabolic demand in the livers of FW milkfish was extremely high. On the other hand, the CS and COX activities in SW-acclimated milkfish were stable under hypothermal stress and the CS:COX activity ratio did not significantly change with temperature. The CS activity and the CS:COX activity ratio corresponded to synergistic interactions between salinity and temperature. Therefore, salinity acclimation significantly affected mitochondrial anabolic demands in milkfish under low-temperature stress.

Several teleost studies used COX activity as an indicator of aerobic metabolism and energy production^5,12,15,41,43^. Therefore, COX activity levels were highly correlated with liver ATP content in various milkfish groups. Under hypothermal stress, ATP levels in FW milkfish livers were reduced whereas in SW milkfish livers they did not significantly change. FW milkfish were less tolerant of hypothermal stress than that by SW milkfish^23^ Reduced ATP production in FW milkfish indicated that they were out of optimal temperature range, underwent metabolic depression, and switched to anaerobic metabolism^2^. Several teleosts compensate for hypothermal stress by enhancing mitochondrial biogenesis and shortening the oxygen and metabolite diffusion distance between the mitochondria and the capillaries in the liver^12,40,43,44^. The mitochondrial densities in milkfish livers were not identified in the present study. Nevertheless, it is known that energy from the TCA cycle was not transferred to the ETC for ATP production in FW milkfish livers. Therefore, the differences in low-temperature tolerance between SW and FW milkfish indicate that salinity must have affected the energy metabolism strategy in their livers under hypothermal stress.

### Anabolic demand for antioxidants under hypothermal stress

Under hypothermal stress, the neutralisation capacity of antioxidant mechanisms participating in cellular physiology was influenced by TCA cycle intermediates^45^. Anabolic demand in the TCA cycle was found in the livers of FW milkfish but not in those of SW milkfish under low-temperature stress. Liver is the primary detoxification organ^46^. Glutathione plays a crucial role in the antioxidant mechanism by neutralising reactive oxygen species (ROS) and participating in redox reactions for antioxidants like glutathione peroxidase, thioredoxin, and peroxiredoxin. Glutathione is a water-soluble tripeptide. The non-essential amino acids glutamate, cysteine, and glycine are glutathione precursors^17,46,47^. Under cold stress, the glutathione and oxidised glutathione (GSSG) levels increased in the liver of the threespine stickleback to neutralise ROS and prevent oxidative damage^48^. Transcriptome analysis revealed that glutathione-related antioxidant mechanisms were upregulated in gilthead seabream under cold stress. In contrast, metabolomics only showed that glutathione metabolism was upregulated^49,50^. In the present study, anabolic demand upregulating glutamate biosynthesis was found in the livers of FW and SW milkfish under hypothermal stress, and FW milkfish has stronger anabolic demand than SW milkfish. Increases in oxidative stress and the expression of the antioxidant protein peroxiredoxin 6 were reported for FW milkfish livers under hypothermal stress. In contrast, SW milkfish under low-temperature stress presented with stable aerobic metabolic rates, anabolic demand, antioxidant responses^31^, and glycogen metabolic rates^24^. FW was more stressful than SW to milkfish livers since that milkfish is a marine euryhaline teleost^23,31^. Our previous studies have also demonstrated that at low temperatures, FW milkfish livers exhibited higher oxidative stress than SW milkfish livers^31^, leading to more anabolic demands in FW individuals to support their antioxidative mechanisms. In addition, the temperature was the major factor for feed consumption according to the same depression pattern in FW and SW milkfish under hypothermal stress^24^. However, milkfish acclimated to FW affect low-temperature tolerance ability and leading to hepatic glycogen breakdown to maintain whole body or liver fuel source^23,24^. In the present study, the mitochondrial energy in FW milkfish livers, in response to increased antioxidant requirements, was directed towards glutamate biosynthesis by the TCA cycle with changes in the ratio of CS/COX. SW milkfish, however, did not change the CS/COX ratio but altered the proportion of biosynthesized amino acids in TCA cycle in the liver to maintain physiological stable upon hypothermal challenge.

### Regulation of genes and proteins correlated with enzyme activities

At 18 °C, the expression levels of the CS gene and protein in the livers of FW milkfish did not significantly change relative to those at 28 °C. Temperature-stable mRNA expression/activity for CS were reported for the livers of cod (*G. morhua*) under cold stress^14^. Insulin treatment in human cells induced CS phosphorylation which, in turn, altered CS activity^51^. Therefore, enzyme activity could be regulated by post-translational modifications like phosphorylation.

On the other hand, the *cox4* mRNA and COX4 protein expression levels were upregulated in FW milkfish livers under low-temperature stress. In contrast, COX activity was downregulated in low-temperature-stressed FW milkfish livers. The COX complex is a large integral membrane protein composed of 13 unique subunits. The COX4 protein has a PKA-mediated phosphorylation site which can be modified to regulate enzyme activity^52^. PGC-1α (peroxisome proliferator-activated receptor-gamma coactivator 1-alpha) is a transcription factor which enhances downstream mRNAs for mitochondrial biogenesis and oxidative phosphorylation (including *cox4*). In several teleosts, it is upregulated with decreasing ambient temperature^12,40,43^. Under hypothermal stress, *cox4* mRNA and COX4 protein expression also increase with decreasing temperature in FW milkfish. Accordingly, these fish should be compensated to maintain constant COX activity under hypothermal stress. However, the COX activity was actually downregulated. Changes in ATP levels induced allosteric modulation regulating COX activity^53^. In the eelpout (*Z. vivparus*), hypothermal stress induced hypoxia, inhibited adenosine, and downregulated COX activity^13^. Cold stress caused a disequilibrium between oxygen supply and oxygen which, in turn, induced anaerobic mitochondrial metabolism^54^. Whereas COX activity is modulated by the oxygen supply in milkfish livers under hypothermal stress COX4 gene and protein expression levels are not. At the same level of hypothermal stress, FW milkfish tend to downregulate aerobic metabolism whereas SW milkfish maintain it.

**Figure 8:**
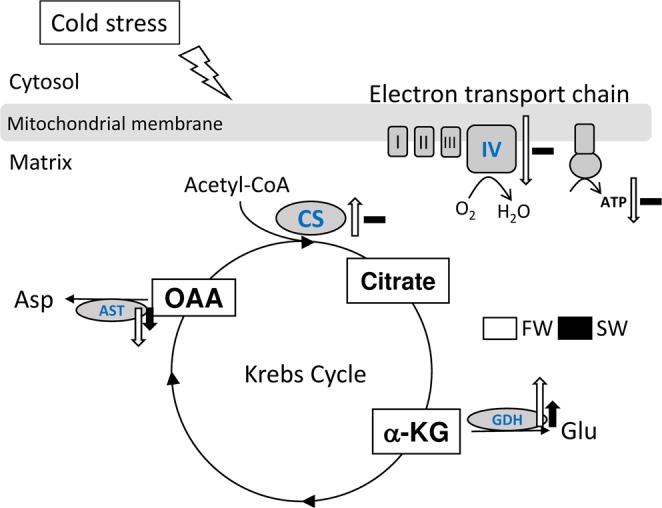
Effects of hypothermal stress on the mitochondria in the livers of freshwater (FW) and seawater (SW) milkfish. AST, aspartate transaminase; CS, citrate synthase; GDH, glutamate dehydrogenase; GLU, glutamate; α-KG, alpha-ketoglutarate; OAA, oxaloacetate.

The present study demonstrated that salinity influences mitochondrial energy flow in milkfish livers under low-temperature stress. Both salinity and environmental temperature affect aerobic metabolism for ATP production and amino acid biosynthesis in the TCA cycle. SW milkfish maintain stable aerobic metabolism under hypothermal stress. In contrast, FW milkfish downregulate aerobic metabolism and redirect energy flow towards the biosynthesis of glutamate which may be used to generate antioxidants (Fig. 8).

## Supplementary information

Supplementary Figure
