# Supplementary material for: Changes in hypothermal stress-induced hepatic mitochondrial metabolic patterns between fresh water- and seawater-acclimated milkfish, Chanos chanos
Source: Sci Rep. 2019 Dec 6;9:18502. doi: 10.1038/s41598-019-55055-4 (PMC6897891; doi:10.1038/s41598-019-55055-4)

Changes in hypothermal stress-induced hepatic mitochondrial metabolic patterns between fresh water- and seawater-acclimated milkfish, *Chanos chanos*

Chia-Hao Chang[1,2], Zong-Zheng Liu[1], Tsung-Han Lee[1,2*]

[1]Department of Life Sciences, National Chung Hsing University, Taichung 402, Taiwan

[2] iEGG and Animal Biotechnology Center, National Chung Hsing University, Taichung 402, Taiwan

Figure S1. Full-length western blot images of (a) CS for figure 3a. (b) β-actin for figure 3a. (c) COX4 for figure 3b. (d) Full-length blot image of β-actin for figure 3b. CS: citrate synthase; COX4: cytochrome c oxidase subunit 4.

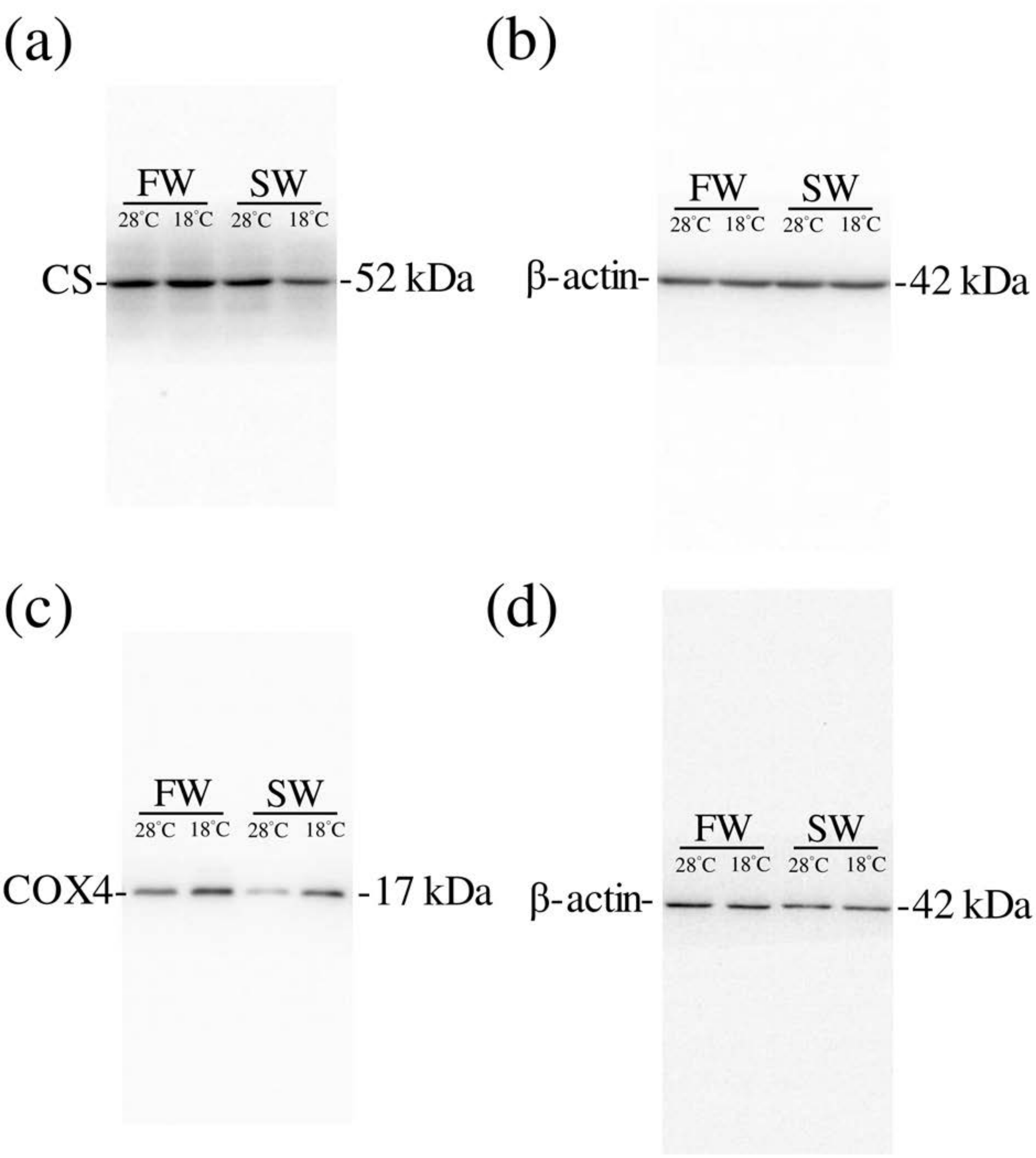

Supplement: Supplementary file 1 — Supplementary Figure [file 41598_2019_55055_MOESM1_ESM.pdf]
